# The Effect of Short-Term NAD3® Supplementation on Circulating Adult Stem Cells in Healthy Individuals Aged 40-70 Years

**DOI:** 10.7759/cureus.55661

**Published:** 2024-03-06

**Authors:** Janine Hellenbrand, Richard J Bloomer, Marie Van der Merwe

**Affiliations:** 1 College of Health Sciences, University of Memphis, Memphis, USA

**Keywords:** supplement, circulating stem cells, nicotinamide adenine dinucleotide (nad+), aging, theacrine

## Abstract

Objective

This study aimed to assess the impact of acute and short-term supplementation with NAD3^®^, a theacrine-containing supplement, on circulating adult stem cell numbers in a healthy male and female population aged 40-70 years.

Methods

This was a double-blind, placebo-controlled crossover study with 12 participants randomized to receive either NAD3® or a placebo for seven days. Blood samples were collected after an overnight fast, before and after the seven-day supplementation period, and one and two hours after the final supplement dose. Using flow cytometry, circulating stem cells, including lymphocytoid CD34^+^ stem cells (CD45^dim^CD34^+^), stem cells associated with vascular maintenance and repair (CD45^dim^CD34^+^CD309^+^), CD34^+^ stem cells linked to a progenitor phenotype (CD45^dim^CD34^+^CD309^neg^), circulating endothelial stem cells (CD45^neg^CD31^+^CD309^+^), and mesenchymal stem cells (CD45^neg^CD90^+^) were quantified.

Results

Acute NAD3^®^ supplementation did not result in the mobilization of stem cells from the bone marrow. However, seven days of daily NAD3^®^ supplementation resulted in selective changes in circulating stem cell numbers. A significant time*treatment interaction was observed for CD45^dim^CD34^+ ^cells (p=0.04) and CD45^dim^CD34^+^CD309^neg ^cells (p=0.04), indicating a decrease in cell numbers with supplementation. There was also a trend toward an increase in circulating endothelial cells (p=0.08) with seven days of NAD3^®^supplementation.

Conclusion

Short-term NAD3® supplementation demonstrated an effect on the quantity of bone marrow-derived stem cells in circulation. The study suggests that this theacrine-containing supplement may play a role in modulating adult stem cell populations, emphasizing the potential impact of NAD3® on regenerative processes. Further research with extended supplementation periods and larger sample sizes is warranted to elucidate the functional consequences of these changes and explore the therapeutic implications for age-related declines in stem cell function.

## Introduction

NAD3® is a commercially available supplement that contains theacrine (1,3,7,9-tetramethyluric acid), cuprous niacin, and Wasabi japonica (a plant similar to horseradish). Theacrine is a major purine alkaloid found in the leaves of wild tea plant species such as Camellia kucha. It is structurally similar to caffeine and has been shown to have various health benefits [1] (e.g., antioxidative [2] and anti-inflammatory properties [3], fatigue reduction [4], and suppressive effects on breast cancer cell metastasis [5]). It also affects cognitive performance and psychometric parameters: the theacrine supplement (TeaCrine®) at a dose of 200 mg/day led to a significant increase in energy, a trend toward improved concentration, and did not affect heart rate (HR) or blood pressure. It is also suggested to improve measures of subjective feelings [6]. Theacrine-containing products have mostly been tested in younger adults [7]; however, in the study by Roberts et al., where the theacrine-containing NAD3® supplement was used, safety was assessed in a population 40-60 years of age [8].

Clinical studies have demonstrated that NAD3® supplementation can improve biomarkers of lipid metabolism [8]. In vitro NAD3® treatment of C2C12 muscle cells also increased cellular nicotinamide adenine dinucleotide (NAD+) levels concomitantly with the upregulation of the enzyme nicotinamide phosphoribosyl transferase (NAMPT) [9]. NAD+ is a coenzyme derived from the vitamin niacin, also known as vitamin B3, and NAMPT is an enzyme that functions in the synthesis of NAD+. NAD+ has been shown to play a critical role in energy metabolism, cellular senescence, and aging [10]. NAD3® administration has also been shown to increase the NAD+:NADH ratio, which serves as a proxy of mitochondrial electron transport chain activity and overall cellular oxidative metabolic capacity, in human peripheral blood mononuclear cells (PBMCs) [8]. The pathway of NAD+ synthesis is highly regulated, and NAD+ levels are known to decrease with age [11,12].

Hematopoiesis and immunity are maintained by self-renewable hematopoietic stem cells (HSCs) located within the bone marrow [13,14]. Adult HSCs are in a quiescent state to prevent DNA damage and depletion of the stem cell pool. Expansion, maintenance, and differentiation of these stem cells are tightly regulated to assure longevity. Adult stem and progenitor cells, including HSCs, mesenchymal stem cells (MSCs), and endothelial progenitor cells (EPCs), also contribute to peripheral tissue repair and rejuvenation. With age, the number and frequency of HSCs in the bone marrow increase; however, there is a reduction in their regenerative capacity and an increase in cellular senescence [15]. Aged HSCs also demonstrate a skewed differentiation potential to the myeloid lineage and decreased differentiation into the lymphoid lineage, resulting in decreased adaptive immunity and an inability of the body to raise an appropriate immune response [16]. Consequently, elderly populations have reduced adaptive immune function, vaccine failure, increased systemic inflammation, and susceptibility to infectious diseases [17]. Even though the number of myeloid cells is increased, their quality is also compromised [18]. Because of the low metabolic rate of HSCs, the mitochondria have not typically been considered critical in restoring function in aged HSCs. However, recent studies suggest an association between NAD+ levels, mitochondrial activities, and HSC longevity in sustaining health and age-related diseases [19]. Various factors contribute to a reduction in HSC functionality. The NAD+ precursor, nicotinamide riboside (NR), has been shown to improve the cellular health of the aged bone marrow and improve the reconstitution potential of aged HSCs, demonstrating its unique role in aging-induced loss of stem cell function [20]. Supplementation with NAD+ precursors can also restore NAD+ levels in various tissues and organ systems and reverse stem cell dysfunction in aged mice [11,21,22]. The restoration of regenerative capabilities appears to occur through the amelioration of mitochondrial dysfunction [23] and suppression of cellular senescence [22].

HSCs and hematopoietic progenitor cells (HPCs) can be mobilized from the bone marrow. Although this process is crucial for the maintenance and rejuvenation of peripheral tissues with aging [24,25], the prognostic value of circulating stem cells is not well defined. It has been shown that levels of circulating stem inversely correlate with the risk of negative health outcomes (e.g., cardiovascular disease), suggesting that a higher number of circulating stem and progenitor cells may improve tissue damage repair [26].

Drapeau et al. have previously demonstrated that the acute consumption of polyphenol-rich extracts from sea buckhorn berries resulted in the rapid and selective mobilization of the HSCs, MSCs, and EPCs in a population between 28 and 70 years of age [27]. The effect of NAD3® supplementation on the mobilization of various stem cell types in an aged population has not yet been investigated. In the current study, we measured levels of circulating stem cells after short-term supplementation and acute consumption of the theacrine-containing supplement, NAD3®, in a non-diseased population of male and female adults aged 40-70 years.

## Materials and methods

Experimental design

**Figure 1:**
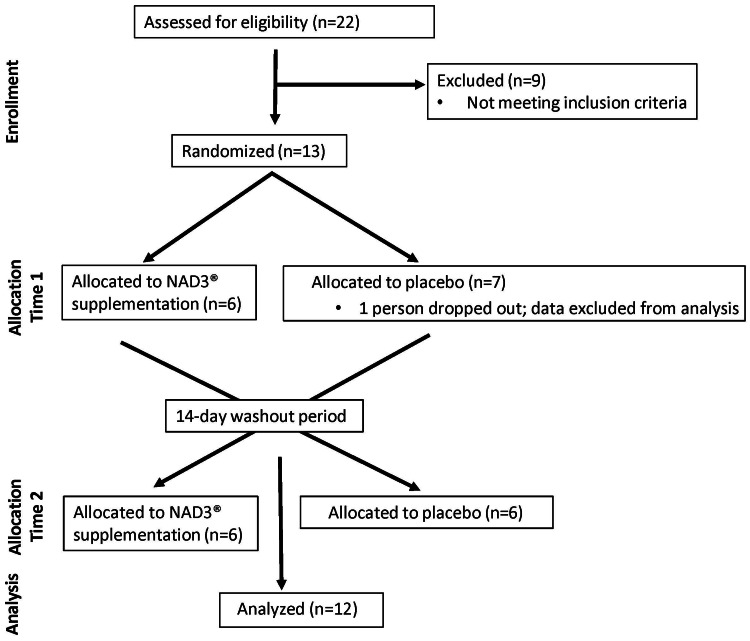
Consort flow diagram.

This study followed a randomized, double-blinded, placebo-controlled, crossover design. The study was registered with ClinicalTrial.gov (NCT02512107). Healthy males and females were recruited via social media. Initial screening was performed using an online screening questionnaire, followed by an onsite screening visit. Twenty-two people provided written consent (IRB-approved protocol #PRO-FY2023-21) and underwent an in-house screening procedure, after which nine people were excluded because of ineligibility. One participant dropped out after the first laboratory visit, and their data were excluded in the final analysis. Twelve subjects completed the study. See Figure 1 for the consort flow diagram of the study design. Participants were healthy men and women between 40 and 70 years of age with vital signs within normal range and no known chronic diseases. Participants had a sedentary to lightly active lifestyle and did not smoke. Participants were then asked to refrain from alcohol consumption and strenuous exercise 48 hours before each laboratory visit.

Study participants completed four laboratory visits. Participants came to the lab after a 10-hour fast for a baseline visit (Day 1) and again after seven days (Day 8) of supplementation (NAD3® or placebo) for each of the study arms. To reduce circadian influences on study outcomes, all study visits occurred between 7 and 10 am, and each participant was scheduled to come to the laboratory at the same time for each laboratory visit. Participants were instructed to avoid stressful situations before the lab visits and to reschedule the visit if they had a stressful situation. No participants had to reschedule. During the baseline visits of each arm, blood was collected in K2-EDTA (dipotassium-ethylene diamine tetraacetic acid) and lithium heparin vacutainers, and test materials (capsules) were distributed. The first capsule was taken with water during the lab visit immediately after blood collection. One capsule was then taken daily for the following six days. After the seventh day (Day 8), participants came to the lab after an overnight fast without consumption of the test material. Similar procedures were followed as mentioned for the baseline visit. After the first blood collection, the test material was consumed, and two additional blood collections then occurred one and two hours after test material consumption.

Study supplement

NAD3® supplement contained the following (per capsule): 344 mg of microcrystalline cellulose, 156 mg of a proprietary blend of Wasabi japonica (freeze-dried rhizome) cultivar, and 97.0% theacrine, copper nicotinic acid chelated complex. Each placebo capsule contained 500 mg of color-matched microcrystalline cellulose.

Measurement of health parameters

The following metabolic parameters were measured using a Piccolo Xpress chemistry analyzer (Abbott Point of Care, Princeton, NJ) at the initial laboratory visit: low-density lipoprotein (LDL), high-density lipoprotein (HDL), triglycerides (TRIG), very low-density lipoproteins (VLDL), aspartate transaminase (AST), alanine transaminase (ALT), cholesterol (CHOL), and glucose (GLU). Insulin was measured using an ELISA assay (Eagle Biosciences, Amherst, NH).

Diet analysis

All participants completed a three-day food log before each laboratory visit. Participants were encouraged to consume a similar diet before each visit. Nutrient analysis was performed using the Food Processor™ software.

Stem cell evaluation by flow cytometry

White blood cell counts were determined at each visit using an Abaxis Vetscan HM5 hematological analyzer (Zoetis, Parsippany, NJ). Within one hour of blood collection, stem cells were stained with fluorescent-labeled antibodies and immediately analyzed by flow cytometry. Replicate samples of 50 µl of heparinized whole blood were stained with the following antibodies to phenotype various stem cell populations: CD45-PacBlue (clone H130, BioLegend, San Diego, CA), CD34-PeCy7 (clone 561, BioLegend, San Diego, CA), CD309-PE (clone A16085H, BioLegend, San Diego, CA), CD31-FITC (clone WM59, BioLegend, San Diego, CA), and CD90-PE (clone 5E10, BioLegend, San Diego, CA). Staining was performed on whole blood using Cal-Lyse® fixation per the manufacturer’s instructions. Briefly, whole blood was incubated with a specific antibody combination for 15 minutes at room temperature in the dark. 50 ul of Cal-Lyse® lysing solution was added and incubated at room temperature for 10 minutes. 0.5 mL of deionized H2O was added, vortexed, and incubated at room temperature for an additional 10 minutes. Samples were then immediately analyzed using an Attune™ flow cytometer (Thermo Fisher Scientific, Waltham, MA). Finally, 500,000 events were collected for each replicate sample. Data were analyzed using Attune™ software and converted to cells/µl of whole blood.

Statistical analysis

All data are mean±SD unless otherwise indicated. Statistical analyses were performed using GraphPad Prism (version 9.5.1; GraphPad Software, San Diego, CA). A repeated measure two-way ANOVA or a fixed-effects model was used to determine potential differences in stem cell populations among treatments. Sidak’s post-hoc analysis was used to detect differences between specific parameters. Statistical significance was accepted at p<0.05.

## Results

This was a randomized, double-blind, placebo-controlled, crossover study. Twelve subjects completed the study. Figure 1 shows the consort flow diagram of the study design. Participants were healthy men and women between 40 and 70 years of age with vital signs within normal range and no known chronic diseases. Participants had a sedentary to lightly active lifestyle and did not smoke. Participants were asked to refrain from alcohol consumption and strenuous exercise 48 hours before each laboratory visit. Twelve participants finished the study, and 100% were compliant in consuming the test material and adhering to the study protocol. The study population included two males and 10 females. The average age and BMI of the study cohort was 50.58±8.45 years and 27.5±4.0 kg/m^2^, respectively. Additional anthropometric data for individual participants are provided in Table 1.

**Table 1:** Anthropometric data of study participants. Anthropometric data of 12 participants collected at the initial visit. Values are mean±SD. (HR, Heart Rate; SBP, systolic blood pressure; DBP, diastolic blood pressure)

Parameter	Baseline (n=12)
Age, years	50.6±8.5
Male	2/12 (16.7%)
Female	10/12 (83.3%)
Height, cm	165.8±10.4
Weight, kg	76.4±16.8
Body mass index, kg/m^2^	27.5±3.9
Resting HR, bpm	74.2±8.9
Resting SBP, mmHg	116.3±8.2
Resting DBP, mmHg	74.3±8.3

Health parameters for the study cohort are provided in Table 2. Lipid, glucose, and liver parameters were all considered to be normal.

**Table 2:** Health parameters of study participants. The health parameters of the 12 participants were collected at the initial visit. Values are mean±SD (ALT, alanine transaminase; AST, aspartate aminotransferase; HDL, high-density lipoprotein; LDL, low-density lipoprotein; VLDL, very low-density lipoprotein)

Parameter	Baseline (n=12)
ALT, U/L	24.2±6.7
AST, U/L	21.8±4.8
Cholesterol, mg/dL	189.6±45.3
Triglycerides, mg/dL	100.6±54.0
HDL, mg/dL	64.0±17.8
LDL, mg/dL	101.6±30.9
VLDL, mg/dL	20.1±10.8
Glucose, mg/dL	101.6±9.7
Insulin, mIU/L	5.5±4.0

Participants were requested to follow their habitual diet during the study period and eat a similar diet in the days before each laboratory visit. No significant differences were detected for total daily calories consumed or macronutrients (protein, carbohydrate, and fat) consumption during either the NAD3® supplementation or placebo arms of the study. As theacrine can interact with adenosine receptors (similar receptors that are blocked by caffeine), caffeine intake was also monitored. There was no significant difference in caffeine consumption detected between lab visits. Nutrient analysis results are shown in Table 3.

**Table 3:** Nutrient consumption before each laboratory visit. Data are mean±SD. Significance is determined with repeated measures two-way ANOVA for time (P1), treatment (P2), and time*treatment interaction (P3).

	NAD3^®^ (n=12)		Placebo (n=12)				
Parameter	Day 1	Day 8	Day 1	Day 8	P1	P2	P3
Kcal/day	1,598±411.1	1,578±502.4	1,490±434.9	1,617±377	0.48	0.83	0.33
Protein (g/day)	69.0±16.0	75.5±26.9	71.1±25.70	65.7±19.0	0.88	0.65	0.12
Carbohydrates (g/day)	171.1±46.1	175.5±67.6	156.6±46.3	186.1±55.6	0.07	0.92	0.17
Fat (g/day)	68.3±25.2	64.2±23.1	63.0±22.6	66.1±21.5	0.91	0.84	0.41
Caffeine (mg/day)	118.4±101.7	152.3±128.3	135.3±109.8	169.2±178.6	0.13	0.74	0.99

To determine if supplementation affected bone marrow function and immune cell differentiation, white blood cell numbers and composition were determined after one week of supplementation. There was no significant change in the absolute number of white blood cells (time*treatment interaction, p=0.51), lymphocytes (time*treatment interaction, p=0.6), monocytes (time*treatment interaction, p=0.98), or neutrophils (time*treatment interaction, p=0.4) numbers. The composition of the white blood cell population was also not altered (p>0.05) with NAD3® supplementation (Table 4).

**Table 4:** Leukocyte absolute number and percentage at baseline and after eight days of supplementation. Data are mean±SD. Significance is determined with repeated measures two-way ANOVA for time (P1), treatment (P2), and time*treatment interaction (P3).

	NAD3^®^ (n=12)		Placebo (n=12)				
Parameter	Day 1	Day 8	Day 1	Day 8	P1	P2	P3
WBC (10^9/L)	5.6±1.7	5.2±1.2	5.5±1.7	5.5±1.2	0.42	0.80	0.51
Lymphocytes (10^9/L)	1.8±2.8	1.7±0.2	1.9±0.3	1.8±0.4	0.23	0.36	0.60
Monocytes (10^9/L)	0.07±0.03	0.08±0.05	0.08±0.03	0.1±0.04	0.20	0.22	0.98
Neutrophils (10^9/L)	3.7±1.6	3.4±1.1	3.6±1.5	3.6±1.1	0.27	0.96	0.40
Lymphocytes (%)	34.2±9.4	34.5±7.9	32.9±13.0	33.7±7.2	0.79	0.75	0.90
Monocytes (%)	1.3±0.5	1.7±0.9	1.5±0.4	1.9±1.0	0.11	0.36	0.90
Neutrophils (%)	64.5±9.3	63.8±8.2	62.3±8.5	64.4±7.9	0.56	0.81	0.27

**Figure 2:**
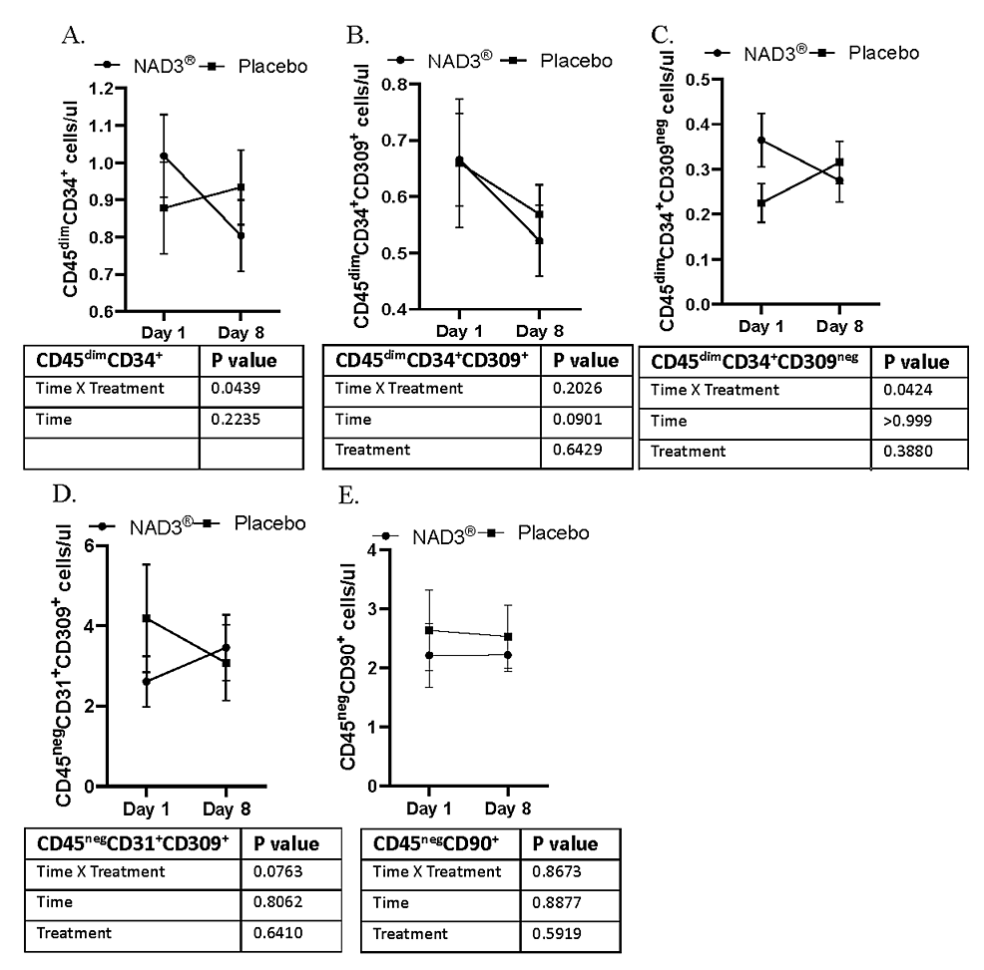
Absolute number of stem cells (cells/ul of blood) measured in whole blood after one week of supplementation with NAD3® or placebo. Line graphs of concentration of various cell populations: A. CD45^dim^CD34^+^, B. CD45^dim^CD34^+^CD309^+^, C. CD45^dim^CD34^+^CD309^-^, D. CD45^-^CD31^+^CD309^+^, and E. CD45^-^CD90^+^, measured in blood at baseline and on day eight of supplementations. Data are mean±SEM, n=12. Significance is determined by repeated measures two-way ANOVA.

We next determined if one week of supplementation influences the absolute number of circulating stem cell populations; lymphocytoid CD34^+^ stem cells (CD45^dim^CD34^+^), stem cells implicated in vascular maintenance and repair (CD45^dim^CD34^+^CD309^+^), CD34^+^ stem cells associated with a progenitor phenotype (CD45^dim^CD34^+^CD309^neg^), circulating endothelial stem cells (CD45^neg^CD31^+^CD309^+^), and mesenchymal stem cells (CD45^neg^CD90^+^) were measured before and after supplementation. A significant time*treatment interaction was detected for CD45^dim^CD34^+^ cells (Figure 2A, p=0.04) and CD45^dim^CD34^+^CD309^neg^ cells (Figure 2C, p=0.04) with no effect on CD45^dim^CD34^+^CD309^+^ cell numbers. Sidak’s post hoc analysis revealed a marginal reduction in CD45^dim^CD34^+^ cells only with NAD3® (p=0.05), not observed with the placebo. Moreover, there was a trend toward a time*treatment interaction in the absolute number of CD45^neg^CD31^+^CD309^+^ (Figure 2D, p=0.08) with increased numbers with supplementation. No significant time*treatment interaction was seen for absolute numbers of CD45^dim^CD34^+^CD309^+^ (p=0.20) and CD45^neg^CD90^+^ cells (p=0.86) (Figures 2B-2E).

**Figure 3:**
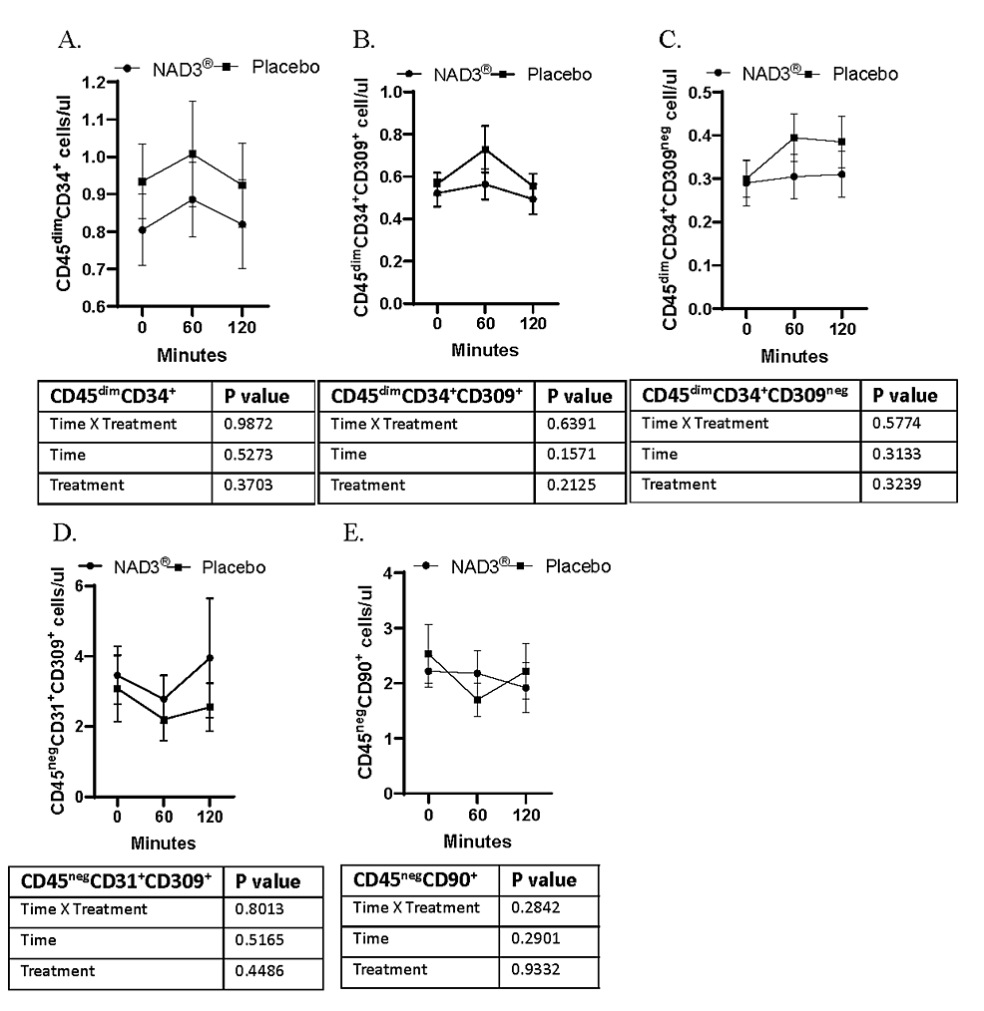
Absolute number of stem cells (cells/ul of blood) measured in whole blood immediately prior to supplement or placebo consumption and one and two hours after consumption. Line graphs of concentration of various cell populations: A. CD45^dim^CD34^+^, B. CD45^dim^CD34^+^CD309^+^, C. CD45^dim^CD34^+^CD309^-^, D. CD45^neg^CD31^+^CD309^+^, and E. CD45^neg^CD90^+^, measured in blood at baseline and after one and two hours of supplement intake. Data are mean±SEM, n=12. Significance is determined by repeated measures two-way ANOVA.

Previous studies have shown acute stem mobilization in response to consumption of a proanthocyanidin-rich extract of sea buckthorn berries. Therefore, we determined the acute effect of NAD3® supplementation on the absolute number of circulating stem cells measured one and two hours after the consumption of the supplement. No time*treatment effect was observed at one and two hours after supplement consumption for CD45^dim^CD34+ (Figure 3A, p=1.0), CD45^dim^CD34+CD309+ (Figure 3B, p=0.64), CD45^dim^CD34+CD309^neg^ (Figure 3C, p=0.58), CD45^neg^CD31+CD309+ (Figure 3D, p=0.80), and CD45^neg^CD90+ cells (Figure 3E, p=0.28).

## Discussion

A central physiological feature of aging is a diminished ability to maintain tissue homeostasis. HSCs have a critical function in this process, and a decline in the capacity of these cells to replenish tissues results in the ineffective repair of damaged tissues [28].

NAD+ has gained considerable attention in the context of aging and particularly its potential role in stem cell longevity. NAD+ levels decrease with age, and compounds that increase NAD+ levels can improve the pool and pluripotency of stem cells [22]. NAD+ precursor compounds (e.g., nicotinamide riboside (NR) and nicotinamide mononucleotide (NMN)) have also been shown to restore aged stem cells through the NAD+/sirtuin pathway and suppress senescence [19,22]. In vitro studies by Mumford et al. demonstrated that the commercially available supplement NAD3® can increase intracellular NAD+ levels and sirtuin activity [9]. Sirtuin proteins are believed to be responsible for the improvement in bone marrow cells resulting from increased NAD. Roberts et al. demonstrated the effect of supplementation with NAD3® on the blood lipid levels and NAD levels in PBMCs [8]. Although no change in NAD+ and NADH levels was observed with supplementation in PBMCs, there was an increase in the NAD+:NADH ratio compared to placebo, indicating that NAD3® can impact the NAD+ metabolome.

Stem cell mobilization, a process by which HSCs are released from the bone marrow and into the bloodstream, is a natural response to various physiological signals and is important for peripheral tissue repair. Potential strategies have been studied to increase stem cell mobilization associated with aging (e.g., dietary interventions (such as caloric restriction, omega-3 fatty acids, and antioxidants), exercise, and hormonal therapies). Drapeau et al. also demonstrated that a polyphenol extract from sea buckthorn berries causes rapid and selective mobilization of specific stem cell types [27].

In the current study, we investigated the effect of NAD3® supplement on the mobilization of various stem cell populations (CD34+ stem cells (CD45^dim^CD34^+^), stem cells implicated in vascular maintenance and repair (CD45^dim^CD34^+^CD309^+^), CD34^+^ stem cells associated with a progenitor phenotype (CD45^dim^CD34^+^CD309^neg^), and circulating endothelial stem cells (CD45^neg^CD31^+^CD309^+^) and mesenchymal stem cells (CD45^neg^CD90^+^) in aging, but otherwise healthy population. No metabolic dysfunction was detected in the study population, and diet and caffeine consumption were consistent throughout the study period.

White blood cell numbers and composition were measured before and after supplementation; however, supplementation did not alter total white blood cell, monocyte, lymphocyte, or neutrophil numbers. Changes in circulating stem cell numbers were also monitored before and after one week of supplementation. NAD3® supplementation resulted in a significant decrease in the CD45^dim^CD34+ population and, specifically, the CD45^dim^CD34^+^CD309^neg^ cells. This population is associated with a progenitor phenotype, while the CD45^dim^CD34^+^CD309^+^ cells contain the transmembrane tyrosine kinase, also known as the vascular endothelial growth factor receptor-2 (VEGFR-2), and it is implicated in vascular maintenance and repair [27].

CD45^dim^CD34^+^CD309^+^ cells were not significantly different from the placebo. The optimal level of circulating progenitor cells is not known; however, it has been suggested that higher baseline numbers are beneficial for peripheral tissue repair. However, during aging, the number and frequency of HSC in the bone marrow of mice and humans increases, but these cells exhibit a concomitant decrease in regenerative capacity [15]. In a mouse model of granulocyte-colony stimulating factor-induced (G-CSF)-induced stem cell mobilization, it was found that the ability to mobilize HSCs was fivefold higher in aged mice because of changes in cell adhesion to bone marrow niche [29]. Therefore, it is possible to speculate that improving stem cell health and reducing senescence in an aged population can reduce progenitor number in circulation.

Interestingly, there was a trend toward an increase in circulating EPCs (CD45^neg^CD31^+^CD309^+^) with NAD3® supplementation. Endothelial stem cells are thought to be involved in blood vessel formation and vascular homeostasis. Depletion of NAD has previously been suggested to contribute to the impairment of EPC mobilization in diabetic conditions, suggesting a potential therapeutic value of NAD in the prevention or treatment of cardiovascular complications of diabetes [30]. The duration of supplementation in this study was limited to one week; therefore, an extended period of supplementation may be necessary to observe a significant change. Nonetheless, these data are intriguing given our relatively small sample size and short period of supplementation.

Acute changes were also determined by monitoring stem cell numbers at one and two hours after supplement ingestion. No effect of NAD3® supplementation was detected on the mobilization of previously described HSCs and progenitor cells. This result suggests that there is no acute effect of NAD3® supplementation on the adhesion of stem cells to the microenvironment within the bone marrow.

Study limitations

There are various limitations in considering the results of this study. The first is that the sample size was small, precluding a more robust statistical analysis. The intervention period of seven days is also relatively short and may not be sufficient to observe the full impact of the supplement. Considering the results, an additional limitation of the current study is that we did not determine the regenerative capability of the circulating stem and progenitor cells to determine changes in the functionality of the cells after supplementation.

## Conclusions

In conclusion, one week of NAD3® supplementation did not cause an acute mobilization of bone marrow-derived stem cells. However, supplementation did result in selective changes in the circulating stem cell numbers; there was a decrease in CD34^+^ progenitor cells and a trend toward an increase in endothelial stem cells. While it is not clear what the functional consequence of these changes is, it does suggest that oral NAD3® supplementation affects the physiology of bone marrow-derived stem cells. Future studies should utilize a longer supplementation period and larger sample sizes to ascertain if NAD3® affects various populations of stem and progenitor cells. Finally, it is essential to assess the regenerative potential of these circulating cells post-supplementation to determine if there are any changes in the functionality of the cells attributable to the intervention.
